# Freezing Hu ram semen: influence of different penetrating cryoprotectants and egg yolk level on the post-thaw quality of sperm

**DOI:** 10.5713/ab.24.0167

**Published:** 2024-06-25

**Authors:** Liuming Zhang, Caiyu Jiang, Xuyang Wang, Tariq Sohail, Yuxuan Sun, Xiaomei Sun, Jian Wang, Yongjun Li

**Affiliations:** 1 Key Laboratory for Animal Genetics and Molecular Breeding of Jiangsu Province, College of Animal Science and Technology, Yangzhou University, Yangzhou 225009, China

**Keywords:** Egg Yolk, Freezing, Hu Ram Semen, Penetrating Cryoprotectant, Sperm Quality

## Abstract

**Objective:**

The Hu sheep is a renowned breed known for its high reproductive rate. It is in estrus all year round, and its breeding population is gradually expanding. However, the current techniques for cryopreserving semen have limited effectiveness, which hinders the continuous development of this species. The purpose of this study is to explore the effects of different penetrating cryoprotectants (CPAs) and egg yolk (EY) concentrations on the cryopreservation of Hu ram semen to determine the most effective combination.

**Methods:**

In this study, the effects of glycerol (GLY), ethylene glycol (EG), dimethylacetamide, dimethyl sulfoxide, different proportions of GLY and EG, EY on sperm quality after thawing were investigated by detecting sperm total motility (TM), progressive motility (PM), straight-line velocity, curvilinear velocity, average path velocity, amplitude of lateral head displacement, wobble movement coefficient, average motion degree, functional integrity (plasma membrane integrity, acrosome integrity) and reactive oxygen species (ROS) level.

**Results:**

When GLY and EG were added together, compared to other concentration groups, 6% GLY significantly (p<0.05) increased TM, PM, plasma membrane integrity, and acrosome integrity of thawed sperm. Additionally, it significantly (p<0.05) decreased the ROS level of sperm. In this study, the TM, PM, and membrane integrity of the 6% EG were significantly (p<0.05) higher than those of the control, 1% GLY+5% EG and 6% GLY+6% EG groups. Compared to other concentration groups, 20% EY significantly (p<0.05) improved the TM, PM, and plasma membrane integrity of thawed sperm. However, the integrity of the acrosome increased with the higher concentration of EY.

**Conclusion:**

In conclusion, the post-thawed Hu ram semen diluted with a diluent containing 6% GLY and 20% EY exhibited higher quality compared to the other groups.

## INTRODUCTION

As an important economic animal in China, the reproductive efficiency of Hu sheep directly impacts the income and sustainable development of the breeding industry. However, the limitations of natural reproduction and the limited resources of breeding stock make artificial insemination (AI) one of the primary methods of sheep reproduction. In the process of AI, preserving and transporting semen is a crucial step and semen cryopreservation technology is an effective method for achieving this task [1].

In the field of animal reproduction, cryopreservation of semen has emerged as a crucial technique for preserving genetic resources, expanding breeding programs, and facilitating assisted reproductive technologies [2]. The development of semen cryopreservation technology provides new possibilities for animal reproduction. It enables the preservation of semen for extended periods and facilitates its transportation over long distances. However, the freezing and thawing process can damage sperm, leading to reduced motility and fertilization capacity of the thawed sperm due to the sensitivity of sperm [3]. Therefore, the cryopreservation of Hu ram semen is a challenging task.

In the process of semen freezing, the selection and use of cryoprotectants (CPAs) are crucial. CPAs mainly consist of permeating and non-permeating agents, with permeating CPAs being primarily utilized in semen cryopreservation at present [4]. Common penetrating CPAs include glycerol (GLY), ethylene glycol (EG), dimethylacetamide (DMA), and dimethyl sulfoxide (DMSO) [5]. The impact of penetrating CPA on sperm quality primarily lies in their ability to penetrate sperm, interact with intracellular water, minimize the formation of intracellular ice crystals, and consequently protect sperm from damage during the freezing process [6].

In addition to CPAs, it is common practice to include egg yolk (EY) in the freezing medium for semen cryopreservation. EY, as a common protective agent, also plays a crucial role in semen cryopreservation [7]. EY is rich in nutrients, lecithin, and low-density lipoprotein (LDL), which can help reduce mechanical damage during freezing [8]. However, the amount of EY used will also affect the freezing effect. Excessive EY can result in an increase in solution viscosity, which can impact sperm motility and the freezing process, while insufficient EY may not offer adequate protection [9].

Although previous studies have investigated the effects of different CPAs and levels of EY on the cryopreservation of semen from various animal species, including Gazella cuvieri [10], dogs [11], and llamas [12]. There is limited information available on the optimal conditions for cryopreserving Hu ram semen. Therefore, the aim of this study is to investigate the effects of different CPAs and EY concentrations on the quality of semen after thawing. This will be achieved by measuring sperm total motility (TM), progressive motility (PM), straight-line velocity (VSL), curvilinear velocity (VCL), average path velocity (VAP), amplitude of lateral head displacement (ALH), wobble movement coefficient (WOB), average motion degree (MAD), plasma membrane integrity, acrosome integrity, and reactive oxygen species (ROS) content. By optimizing the cryopreservation conditions, this study aims to provide a theoretical basis and practical guidance for the cryopreservation technology of semen from Hu rams. This research also aims to contribute to the conservation of genetic resources and the enhancement of reproductive efficiency in this species.

## MATERIALS AND METHODS

### Ethics statement

All animal procedures were carried out following the Animal Ethics Committee of Yangzhou University (ID: 202206132).

### Experimental design

In experiment I, the purpose of this study was to investigate the impact of four different CPAs with membrane-permeable properties including GLY (0%, 2%, 4%, 6%, 8%), EG (0%, 2%, 4%, 6%, 8%), DMA (0%, 2%, 4%, 6%, 8%), DMSO (0%, 2%, 4%, 6%, 8%) on the sperm motility parameters (TM and PM) and biokinetic characteristics (VSL, VCL, VAP, ALH, WOB, and MAD) of Hu ram semen after cryopreservation. Secondly, based on the previous results, the study aimed to investigate the impact of different combinations of CPAs (0%, 6% GLY, 6% EG, 5% GLY+1% EG, 1% GLY+5% EG, 6% GLY+6% EG) on sperm motility parameters (TM and PM), biokinetic characteristics (VSL, VCL, VAP, ALH, WOB, and MAD), membrane integrity, acrosome integrity and ROS level after cryopreservation.

In experiment II, the purpose of this study was to investigate the impact of different concentration of EY (0%, 10%, 20%, 30%, 40%, 50%) on sperm motility parameters (TM and PM), biokinetic characteristics (VSL, VCL, VAP, ALH, WOB, and MAD), membrane integrity, acrosome integrity and ROS level after cryopreservation.

### Animals management and collecting sperm

Five healthy breeding Hu rams (2 to 3 years old) with proven fertility were housed at the Yangzhou University sheep facility from September to November 2023. The Hu sheep exhibit estrus throughout the year. The rams were managed under similar intensive conditions during this research. Specifically, the rams were fed a designated amount of concentrate and alfalfa grass daily, and they had unrestricted access to water.

A total number of 45 ejaculates (9 ejaculates per ram) were collected from five rams using an artificial vagina twice a week. Semen samples were taken back to the laboratory within 30 min and immersed in a 37°C water bath for semen quality evaluation. Samples were evaluated and entered the experiment if the following standards were met: volume of 0.5 to 1.5 mL, minimum sperm concentration of 2.5×10^9^/mL and TM ≥80%. On each day for the semen collection, only semen samples that meet the standards were pooled to balance the sperm concentration, reduce individual differences, and used in subsequent experiments. All collected ejaculates were utilized for the research.

### Cryoprotective agents and diluents

In the experiment, the Tris-based (Tris 3.64 g/100 mL, citric acid 1.82 g/100 mL, fructose 0.5 g/100 mL, sodium penicillin and streptomycin sulfate 20,000 IU/100 mL) semen diluent was used. In the experiment I, diluent I was composed of 80% (*v/v*) Tris-based diluent and 20% (*v/v*) EY. According to the experimental design, diluent II was composed of CPAs (GLY, EG, DMA, DMSO) with different concentrations and dilution I.

In the experiment II, diluent I was composed of Tris-based diluent and EY with different concentrations according to the experimental design. Diluent II contained 94% (v/v) diluent I and 6% (v/v) GLY.

### General protocol for freezing and thawing

Pooled semen samples were divided into equal aliquots and diluted with diluent I in a 1:3 ratio at 37°C. Afterward, the samples were equilibrated at 4°C for 2.5 h. Subsequently, the samples were diluted with diluent II in a 1:2 ratio at 4°C. Afterward, the samples were equilibrated at 4°C for 2.5 h. Following equilibration, the diluted samples were loaded into 0.25 mL straws and sealed with sealing powder. The straws were frozen in liquid nitrogen vapor, 4 cm above the liquid nitrogen, for 20 min. Subsequently, the straws were immediately plunged into the liquid nitrogen for storage.

After being stored for one week, the frozen straws were randomly selected and thawed individually at 55°C for 8 s in a water bath for further evaluation.

### Sperm motility parameters and biokinetic characteristics analysis

The sperm motility parameters (TM and PM) and biokinetic characteristics (VSL, VCL, VAP, ALH, WOB, and MAD) were assessed immediately after thawing using a computer-assisted sperm analyzer (CASA, ML-608JZ II; Mailang, Nanning, China). Thawed samples were diluted at a ratio of 1:4.5 with a Tris-based diluent and then incubated at 37°C for 3 min. For evaluation, a 1.4 μL drop of the sample was placed on a pre-warmed (37°C) MACRO sperm counting chamber (YA-1; Yucheng, Nanjing, China). A minimum of 500 sperm were counted under a phase contrast microscope (ML-800; Mailang, China) equipped with a CCD-camera (MD06200C; Mailang, China) at 37°C and 100× magnification.

### Assessment of sperm plasma membrane integrity

The hypo-osmotic swelling test (HOST) was used to evaluate sperm membrane integrity based on the curled tails. It was performed by incubating a 20 μL aliquot of thawed semen with 200 μL hypo-osmotic solution (9 mg/mL fructose and 4.9 mg/mL sodium citrate dissolved into water) in a water bath (37°C) for 30 min. After incubation, 1.8 μL of the mixture was spread using a MACRO sperm counting chamber (YA-1; Yucheng, China). Membrane integrity was assessed by counting 200 sperm under a phase-contrast microscope (CX31; Olympus, Tokyo, Japan) at 400× magnification.

### Assessment of sperm acrosome integrity

Sperm acrosome integrity was assessed using fluorescein isothiocyanate-conjugated peanut agglutinin (FITC-PNA) combined with propidium iodide (PI) staining. Briefly, the thawed samples were diluted 1:6 with a Tris-based diluent and incubated by mixing 100 μL semen samples with 2 μL PI (0.5 mg/mL) and FITC-PNA (200 μg/mL) in a water bath (37°C) for 10 min in the dark. After incubation and the addition of 700 μL phosphate-buffered solution (PBS), the mixture was analyzed by a FACS caliber flow cytometer (Beckman Coulter, Shanghai, China). Sperm with FITC^−^/PI^−^ and FITC^−^/PI^+^ were considered to have intact acrosomes, and 10,000 sperm were counted using flow cytometry.

### Assessment of sperm reactive oxygen species level

Sperm ROS level was measured using the 2,7-dichlorodi-hydrofluorescein diacetate (DCFH-DA) probe. First, the thawed semen samples were diluted 1:6 with a Tris-based diluent. The dilution process involved incubating 50 μL semen samples with 2 μL DCFH-DA (10 mM) at a water bath (37°C) for 30 min in the dark. After incubation, the samples were washed with PBS and resuspended by adding 400 μL PBS. The fluorescence intensity (488 nm excitation and 525 nm emission) was measured using a multi-mode microplate reader (PerkinElmer, Waltham, MA, USA) and indicated the ROS level.

### Statistical analysis

Statistical analyses were conducted using IBM SPSS 25.0 (SPSS Inc., Chicago, IL, USA). The Shapiro-Wilk test was conducted to determine if the data conforms to a normal distribution. The data showed a normal distribution and was analyzed using one-way analysis of variance. The means were examined by Tukey HSD test, with significance set at p<0.05. All data were expressed as mean±standard error of the mean, and each group was replicated four times.

## RESULTS

### Effect of glycerol on sperm motility parameters and biokinetic characteristics after cryopreservation

As shown in Table 1, the frozen-thawed sperm TM, PM, and MAD of the 6% GLY group were significantly higher (p<0.05) than those of the other groups. The frozen-thawed sperm VSL of the control group was lowest (p<0.05) compared with other GLY groups and there was no difference (p>0.05) in sperm VSL between the GLY groups. The frozen-thawed sperm VCL, VAP, and ALH of the 6% GLY group were significantly higher (p<0.05) than those of the control and 8% groups and there were no significant difference (p>0.05) with the 2%, 4%, 6% GLY groups.

### Effect of ethylene glycol for sperm motility parameters and biokinetic characteristics after cryopreservation

The frozen-thawed sperm TM and PM of the 6% EG group were significantly higher (p<0.05) than those of the other groups, as shown in Table 2. The frozen-thawed sperm VSL of the 6% EG group was significantly higher than that of the other groups (p<0.05) except the 2% EG group (p>0.05). The frozen-thawed sperm VCL, VAP, and ALH of the 6% EG group were significantly higher (p<0.05) than those of the control and 4% groups. The frozen-thawed sperm WOB of the control group was lowest than that of the other EG groups. The frozen-thawed sperm MAD of the 6% EG group was significantly higher than that of the other groups (p< 0.05) except the 4% EG group (p>0.05).

### Effect of dimethyl sulfoxide for sperm motility parameters and biokinetic characteristics after cryopreservation

As shown in Table 3, the frozen-thawed sperm TM, PM, and all biokinetic characteristics did not show significant difference (p>0.05) between all the groups.

### Effect of dimethylacetamide for sperm motility parameters and biokinetic characteristics after cryopreservation

The frozen-thawed sperm TM, PM, and MAD of the 2% DMA group were significantly higher (p<0.05) than those of the other groups, as shown in Table 4. The frozen-thawed sperm VSL, VCL, VAP, and ALH of the 2%, 4% DMA groups were significantly higher (p<0.05) than those of the other groups. There was no difference (p>0.05) in the frozen-thawed sperm WOB between the groups.

### Effect of cryoprotectants combination for sperm motility parameters and biokinetic characteristics after cryopreservation

As shown in Table 5, the frozen-thawed sperm TM, PM, VCL, VAP, ALH, and MAD of the 6% GLY group were significantly higher (p<0.05) than those of the other groups. The frozen-thawed sperm VSL of the 6% GLY group was significantly higher (p<0.05) than that of the control, 1% GLY+5% EG, 6% GLY+6% EG and there were no significant difference (p>0.05) compared with the 6% EG and 5% GLY +1% EG. The frozen-thawed sperm WOB of the control group was lowest (p<0.05) compared with other groups.

### Effect of cryoprotectants combination for sperm plasma membrane and acrosome integrity after cryopreservation

**Figure 1:**
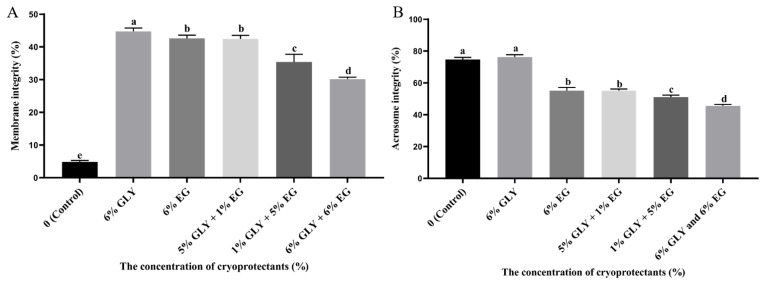
Effect of cryoprotectants (CPAs) combination on Hu ram sperm parameters post-thawing. (A) Plasma membrane integrity as the percentage of sperm with curled tail after hypo-osmotic swelling test (HOST) test. (B) Acrosome integrity as the percentage of sperm with intact acrosome. ^a–e^ Different letter indicates significant differences (p<0.05), the same letter indicates no significant differences (p>0.05). GLY, glycerol; EG, ethylene glycol.

The frozen-thawed sperm membrane integrity of the 6% GLY group was highest (p<0.05) than that of the other groups, as shown in Figure 1A. The frozen-thawed sperm membrane integrity of the 6% EG and 5% GLY+1% EG was significantly higher (p<0.05) than that of the control, 1% GLY+5% EG, 6% GLY+6% EG. As shown in Figure 1B, the frozen-thawed sperm acrosome integrity of the control and 6% GLY was significantly higher (p<0.05) than that of the other groups. The frozen-thawed sperm acrosome integrity of the 6% EG and 5% GLY+1% EG was significantly higher (p<0.05) than that of the 1% GLY+5% EG and 6% GLY+6% EG.

### Effect of cryoprotectants combination for sperm reactive oxygen species level after cryopreservation

**Figure 2:**
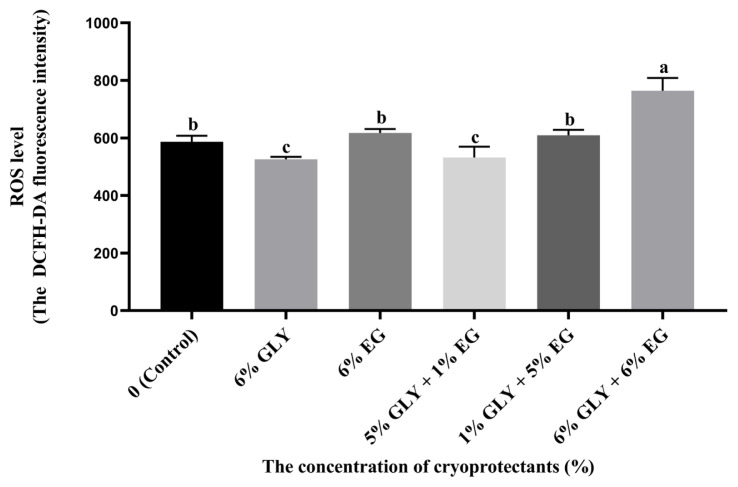
Effect of cryoprotectants (CPAs) combination on Hu ram sperm ROS level post-thawing. ^a–c^ Different letter indicates significant differences (p<0.05), the same letter indicates no significant differences (p>0.05). GLY, glycerol; EG, ethylene glycol; ROS, reactive oxygen species; DCFH-DA, 2,7-dichlorodi-hydrofluorescein diacetate.

As shown in Figure 2, the frozen-thawed sperm ROS level of the 6% GLY and 5% GLY+1% EG was significantly lower (p<0.05) compared with other groups. The frozen-thawed sperm ROS level of the control, 6% EG and 1% GLY+5% EG was significantly lower (p<0.05) than that of the 6% GLY+ 6% EG group.

### Effect of egg yolk level for sperm motility parameters and biokinetic characteristics after cryopreservation

The frozen-thawed sperm TM, PM, and MAD of the 20% EY group were significantly higher (p<0.05) than those of the other groups, as shown in Table 6. The frozen-thawed sperm VSL, VCL, VAP, and ALH of the control, 10%, 20% EY groups were significantly higher (p<0.05) compared with other groups. The frozen-thawed sperm WOB of the 20% EY group was significantly higher than that of the 40% and 50% EY groups.

### Effect of egg yolk level for sperm plasma membrane and acrosome integrity after cryopreservation

**Figure 3:**
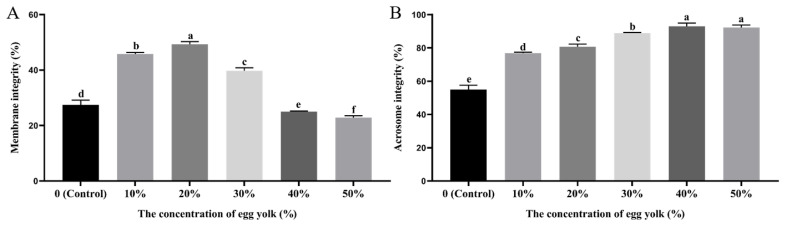
Effect of egg yolk (EY) level on Hu ram sperm parameters post-thawing. (A) Plasma membrane integrity as the percentage of sperm with curled tail after hypo-osmotic swelling test (HOST) test. (B) Acrosome integrity as the percentage of sperm with intact acrosome. ^a–f^ Different letter indicates significant differences (p<0.05), the same letter indicates no significant differences (p>0.05).

As shown in Figure 3A, the frozen-thawed sperm membrane integrity of the 20% EY group was highest (p<0.05) compared with other groups. The frozen-thawed sperm membrane integrity of the 10% EY group was significantly higher than that of the other groups (p<0.05) except 20% EY group (p>0.05). The frozen-thawed sperm membrane integrity of the 30% EY group was significantly higher (p<0.05) than that of the control, 40% and 50% EY groups. The frozen-thawed sperm acrosome integrity of the control group was lowest (p<0.05) compared with other groups, as shown in Figure 3B. The frozen-thawed sperm acrosome integrity of the 40% and 50% EY groups was highest (p<0.05) than that of the other groups. And the acrosome integrity gradually increased with the increase of EY concentration.

## DISCUSSION

Semen cryopreservation involves semen dilution, cooling equilibration, freezing, and thawing. During the cooling equilibration, sperm are susceptible to cooling damage or cold shock due to membrane damage caused by lipid transitioning from a liquid state to a gel state [13]. In the process of freezing and thawing, sperm are prone to forming ice crystals at 0°C to −60°C due to the slow rate of cooling or heating [14]. Due to the movement of ice crystals and the continuous increase in volume, ice crystals can exert mechanical pressure on sperm, leading to the destruction of sperm membranes and organelles. Theoretically, different penetrating CPAs, along with lecithin and LDL contained in EY, can potentially minimize the harm caused by cold shock and ice crystals during the process of freezing and thawing semen. Therefore, this experiment explored the effects of various penetrating CPAs and EY on the cryopreservation of Hu ram semen. The results showed that 6% GLY was the most suitable penetrating CPAs for cryopreservation, and the optimal addition of EY was 20%.

In semen cryopreservation, the addition of CPAs to the diluent plays a crucial role in preventing ice crystallization as it can minimize sperm damage during freezing. As shown in Table 1, adding GLY to the diluent can significantly improve sperm motility after thawing compared to the control group. At the same time, with the increase in GLY concentration the sperm motility after thawing exhibited a trend of initially increasing and then decreasing, with the most significant effect observed at a concentration of 6%. GLY is the most used CPA in animal semen cryopreservation, which can penetrate the cell membrane and enter the sperm, displacing the water within the cell [15]. This process partially dehydrates the cell, thereby preventing the formation of ice crystals. At the same time, the high concentration of GLY has more toxic side effects on sperm, which may be attributed to its osmotic and chemical toxicity, leading to the destabilization of the sperm membrane [16]. Consequently, this results in decreased sperm motility after thawing. Fernández-Santo et al [17] research on Iberian Red Deer also showed that the effect of a 6% GLY concentration on sperm cryopreservation was better than that of 3% and 12%. The result was similar to those of the study, indicating that the optimal concentration of GLY has the most significant impact on semen cryopreservation. Strzeżek and Reksa [11] used a glycerol concentration of 4% when freezing dog semen. This difference in the experiment may be attributed to variations in species and freezing techniques. The molecular weight of GLY is relatively large, resulting in a slow permeation rate and a greater toxic effect. Therefore, other penetrating CPAs have been tried to replace GLY in semen cryopreservation. Theoretically, EG has a lower molecular weight, higher permeability, and lower osmotic pressure, making it more effective for semen cryopreservation compared to GLY. As shown in Table 2, EG exhibited a similar trend to GLY, with the most effective semen cryopreservation achieved at 6%. Santiani et al [18] demonstrated that GLY and EG can produce similar freezing effects in the cryopreservation of alpaca semen. Acha et al [19] used 1% EG in freezing Andalusian donkey semen and achieved better freezing results. This difference may be caused by variations among species. DMSO is a sulfur-containing organic compound with extremely low toxicity and has been widely used as a cell CPA and membrane penetrant [20]. Studies have shown that the application effect of DMSO varies significantly among different species and cell types [21]. DMSO is considered the most effective CPA in fish sperm cryopreservation [22]. Kundu et al [23] study showed that DMSO had a positive effect on the cryopreservation of goat semen. However, when DMSO is used as a CPA, the sperm of Hu rams basically dies after thawing, as shown in Table 3. This difference may be attributed to variations in the sperm membrane structure and fluidity among different species, indicating that DMSO may not be suitable for cryopreserving Hu ram semen [24]. Compared with GLY, amides contain nitrogen-containing functional groups and methyl groups, making them highly hydrophilic and can penetrate sperm membranes smoothly, thereby reducing the formation of intracellular ice crystals [25]. Therefore, DMA theoretically has a better cryopreservation effect than GLY. Kashiwazaki et al [26] found that DMA played a significant role in the cryopreservation of rabbit semen. In this study, only 2% DMA can be used for the cryopreservation of Hu ram semen, but when compared with other CPAs, DMA shows a poor effect. This may also be caused by differences between species.

These low molecular weight CPAs can easily penetrate the cell membrane and enter the interior of sperm. During the freezing process, cells can maintain a balance in osmotic pressure inside and outside the cell, minimize the loss of water from inside to outside of the cell, and prevent excessive dehydration of the cell [27]. At the same time, they can also reduce the concentration of electrolytes in the unfrozen solution within cells and protect sperm from damage caused by high concentrations of electrolytes [28]. These effects collectively enhance sperm tolerance to freezing and improve motility after thawing. As can be seen from Table 5, the sperm TM of the 6% GLY group after thawing is significantly higher than that of the 6% EG group and other groups with additional combined substances. This shows that 6% GLY is the most suitable for the cryopreservation of Hu ram semen. Moreover, when 6% GLY and 6% EG were combined, the toxic effect on sperm significantly increased, resulting in a decrease in the integrity of the plasma membrane and acrosome of thawed sperm. This may be because the high concentration of CPAs has altered the osmotic pressure of sperm, leading to sperm damage [29]. Gonzalez-Castro et al [30] found in the study of stallion sperm that the combination of two CPAs has a better effect than using a single CPA. These differences may be due to the type of dilution, freezing protocols, and sperm ram freezability [31]. During the process of sperm freezing and thawing, a significant amount of ROS is generated due to dilution, changes in osmotic pressure, cooling equilibration, and cold shock [32]. Proper concentration of ROS is involved in regulating several important signaling reactions and maintaining the integrity of sperm structure [33]. Excessive ROS can lead to lipid peroxidation of sperm, disrupt the integrity of the plasma membrane, and impair mitochondrial function. This can result in inadequate adenosine triphosphate production, ultimately diminishing the biokinetic characteristics and viability of sperm [34]. In this study, the group treated with 6% GLY group reduced the level of ROS in sperm. This reduction may be attributed to the ability of GLY at this concentration to minimize the formation of ice crystals, consequently lowering the oxidative stress response.

In addition, EY is a common component in semen frozen diluent, and its primary function is to protect the sperm plasma membrane from cold shock. When mammalian sperm are cooled from body temperature to freezing temperature, it results in reduced sperm motility and carbohydrate metabolism, leading to the release of intracellular enzymes and lipids [35]. Related research shows that this harmful effect can be reduced by adding an appropriate concentration of yolk in the diluent [36]. As shown in Table 6, the motility of thawed sperm initially increased and then decreased with the rise in EY concentration, peaking at 20%. At the same time, compared with the control group, 40% and 50% EY had a negative impact. In the study of Bakhtiari rams [37], it was found that 20% yolk had the best semen cryopreservation effect, which was consistent with the results obtained in this study. Garde et al [10] found that using a Tris-based dilution, 5% EY was more effective than 20% in freezing Gazella cuvieri semen. The difference between this and the results of the present study may be due to variations in dilution methods and species. The substances that play a role in EY are usually considered to be LDL and lecithin. Studies have shown that these two substances can stabilize cell membranes, replace phospholipids lost during freezing and thawing, and prevent cholesterol and phospholipids from leaking out [38]. However, the negative effect of a high concentration of EY may be attributed to the increase in diluent viscosity resulting from the addition of a large amount of EY, which inhibits sperm respiration and reduces sperm motility [39]. The plasma membrane and acrosome are crucial structures in sperm, playing essential roles in sperm capacitation, acrosome reaction, and maintaining the osmotic balance of cells [40]. In this study, the acrosome integrity rate increased with the increase in EY concentration. This phenomenon may be attributed to the beneficial substances in EY adhering to the surface of sperm, thereby protecting the acrosome integrity.

## CONCLUSION

In conclusion, the results of this study indicate that a Tris-based diluent containing 20% EY and 6% GLY significantly improves post-thaw Hu ram sperm parameters. In addition, our study provided valuable insights into the effect between CPAs, EY concentrations, and sperm quality in Hu ram semen cryopreservation. By optimizing these factors, we could potentially enhance the reproductive efficiency of this important breed, contributing to its sustainable development and economic prosperity. Check around here.

## Figures and Tables

**Table 1:** Effect of GLY for sperm motility parameters and biokinetic characteristics

GLY (%)	TM (%)	PM (%)	VSL (μm/s)	VCL (μm/s)	VAP (μm/s)	ALH (μm)	WOB (%)	MAD (°/s)
0 (Control)	3.30±0.71^e^	2.20±0.51^e^	19.15±3.55^b^	34.65±3.39^c^	24.50±2.39^c^	10.15±0.99^c^	0.58±0.08	3.19±0.21^d^
2	22.85±1.98^d^	15.05±1.5^d^	39.57±1.22^a^	59.52±2.87^ab^	42.09±2.03^ab^	17.43±0.84^ab^	0.53±0.03	10.19±0.90^c^
4	55.22±1.27^b^	42.30±1.09^b^	41.06±0.73^a^	60.65±0.87^ab^	42.88±0.61^ab^	17.76±0.25^ab^	0.51±0.03	25.48±2.20^b^
6	67.88±1.37^a^	50.13±0.15^a^	41.18±0.53^a^	62.58±0.48^a^	44.25±0.34^a^	18.33±0.14^a^	0.55±0.02	34.75±1.30^a^
8	48.43±1.93^c^	34.92±1.18^c^	37.46±0.86^a^	55.66±1.65^b^	39.35±1.17^b^	16.30±0.49^b^	0.55±0.02	22.78±1.20^b^

GLY, glycerol; TM, total motility; PM, progressive motility; VSL, straight-line velocity; VCL, curvilinear velocity; VAP, average path velocity; ALH, amplitude of lateral head displacement; WOB, wobble movement coefficient; MAD, average motion degree.

a–e Means within a column with different superscripts differ significantly (p<0.05).

**Table 2:** Effect of EG for sperm motility parameters and biokinetic characteristics

EG (%)	TM (%)	PM (%)	VSL (μm/s)	VCL (μm/s)	VAP (μm/s)	ALH (μm)	WOB (%)	MAD (°/s)
0 (Control)	2.64±0.70^e^	0.86±0.16^e^	33.24±0.62^b^	42.08±2.59^c^	29.76±1.83^c^	12.32±0.76^c^	0.08±0.08^b^	0.63±0.04^d^
2	19.30±1.21^d^	12.03±0.69^d^	34.45±0.41^ab^	54.05±1.60^ab^	38.22±1.13^ab^	15.83±0.47^ab^	0.55±0.03^a^	10.36±0.74^c^
4	50.02±0.47^b^	32.08±1.05^b^	31.70±1.17^b^	49.98±0.88^b^	35.34±0.63^b^	14.64±0.26^b^	0.60±0.03^a^	22.47±2.02^ab^
6	55.45±1.18^a^	38.94±1.26^a^	36.98±1.10^a^	58.36±0.67^a^	41.27±0.47^a^	17.09±0.20^a^	0.63±0.03^a^	25.33±0.74^a^
8	41.37±0.34^c^	27.53±0.86^c^	33.52±1.39^b^	53.78±0.95^ab^	38.03±0.68^ab^	15.75±0.28^ab^	0.66±0.05^a^	20.92±1.18^b^

EG, ethylene glycol; TM, total motility; PM, progressive motility; VSL, straight-line velocity; VCL, curvilinear velocity; VAP, average path velocity; ALH, amplitude of lateral head displacement; WOB, wobble movement coefficient; MAD, average motion degree.

a–e Means within a column with different superscripts differ significantly (p<0.05).

**Table 3:** Effect of DMSO for sperm motility parameters and biokinetic characteristics

DMSO (%)	TM (%)	PM (%)	VSL (μm/s)	VCL (μm/s)	VAP (μm/s)	ALH (μm)	WOB (%)	MAD (°/s)
0 (Control)	5.16±2.00	2.11±1.09	28.04±10.21	47.85±10.54	33.83±7.45	14.01±3.08	0.64±0.25	3.44±0.21
2	3.65±0.79	2.44±0.68	39.79±2.44	56.65±2.59	40.05±1.83	16.59±0.76	0.39±0.18	1.87±0.81
4	4.34±0.18	2.77±0.44	42.19±0.57	55.09±0.36	38.96±0.26	16.14±0.11	0.50±0.14	2.09±0.32
6	4.68±0.93	1.94±0.76	42.43±3.90	64.52±3.29	45.62±2.33	18.90±0.96	0.44±0.11	2.84±0.94
8	4.82±0.95	2.53±0.54	32.86±4.49	54.83±5.29	38.77±3.75	16.06±1.55	0.49±0.09	3.15±0.27

DMSO, dimethyl sulfoxide; TM, total motility; PM, progressive motility; VSL, straight-line velocity; VCL, curvilinear velocity; VAP, average path velocity; ALH, amplitude of lateral head displacement; WOB, wobble movement coefficient; MAD, average motion degree.

**Table 4:** Effect of DMA for sperm motility parameters and biokinetic characteristics

DMA (%)	TM (%)	PM (%)	VSL (μm/s)	VCL (μm/s)	VAP (μm/s)	ALH (μm)	WOB (%)	MAD (°/s)
0 (Control)	1.91±0.55^cd^	1.15±0.36^c^	30.00±1.17^b^	41.26±3.32^b^	29.18±2.35^b^	12.09±0.97^b^	0.38±0.22	1.85±0.43^b^
2	35.55±2.32^a^	23.67±1.62^a^	44.78±2.29^a^	62.54±4.26^a^	44.22±3.01^a^	18.31±1.25^a^	0.44±0.05	12.41±2.21^a^
4	8.09±2.23^b^	6.14±1.12^b^	43.19±0.90^a^	59.29±0.63^a^	41.92±0.44^a^	17.36±0.18^a^	0.36±0.01	3.74±0.57^b^
6	6.62±0.12^bc^	4.89±0.48^b^	17.17±1.02^c^	25.73±1.80^c^	18.19±1.28^c^	7.54±0.53^c^	0.35±0.01	2.84±0.07^b^
8	1.34±1.02^d^	0.67±0.38^c^	9.50±5.08^c^	16.76±8.77^c^	11.85±6.21^c^	4.91±2.57^c^	0.33±0.17	0.59±0.30^b^

DMA, dimethylacetamide; TM, total motility; PM, progressive motility; VSL, straight-line velocity; VCL, curvilinear velocity; VAP, average path velocity; ALH, amplitude of lateral head displacement; WOB, wobble movement coefficient; MAD, average motion degree.

a–d Means within a column with different superscripts differ significantly (p<0.05).

**Table 5:** Effect of CPAs combination for sperm motility parameters and biokinetic characteristics

CPA (%)	TM (%)	PM (%)	VSL (μm/s)	VCL (μm/s)	VAP (μm/s)	ALH (μm)	WOB (%)	MAD (°/s)
0 (Control)	3.24±1.67^e^	1.43±0.59^f^	11.28±2.24^c^	15.72±2.56^d^	11.12±1.81^d^	4.61±0.75^d^	0.25±0.05^b^	0.98±0.27^c^
6% GLY	73.19±2.32^a^	57.84±0.99^a^	42.02±1.63^a^	63.87±1.23^a^	45.16±0.87^a^	18.71±0.36^a^	0.56±0.04^a^	40.92±4.50^a^
6% EG	61.03±0.62^b^	44.94±0.42^c^	39.14±0.40^ab^	58.72±0.85^b^	41.53±0.60^b^	17.20±0.25^b^	0.58±0.02^a^	27.72±0.97^b^
5% GLY+1 % EG	61.99±1.92^b^	48.98±0.74^b^	39.35±0.83^ab^	59.08±1.41^b^	41.78±0.99^b^	17.30±0.41^b^	0.51±0.02^a^	30.60±2.97^b^
1% GLY+5% EG	49.28±1.36^c^	35.18±1.43^d^	37.04±1.09^b^	55.80±0.75^bc^	39.46±0.53^bc^	16.35±0.22^bc^	0.55±0.01^a^	24.19±1.98^b^
6% GLY+6% EG	17.68±1.58^d^	11.26±0.99^e^	35.75±0.78^b^	51.67±1.72^c^	36.53±1.21^c^	15.13±0.50^c^	0.46±0.06^a^	7.95±0.69^c^

CPA, cryoprotectant; TM, total motility; PM, progressive motility; VSL, straight-line velocity; VCL, curvilinear velocity; VAP, average path velocity; ALH, amplitude of lateral head displacement; WOB, wobble movement coefficient; MAD, average motion degree.

a–f Means within a column with different superscripts differ significantly (p<0.05).

**Table 6:** Effect of EY level for sperm motility parameters and biokinetic characteristics

EY (%)	TM (%)	PM (%)	VSL (μm/s)	VCL (μm/s)	VAP (μm/s)	ALH (μm)	WOB (%)	MAD (°/s)
0 (Control)	35.67±2.73^d^	28.72±1.97^d^	42.68±1.28^a^	64.10±1.34^a^	45.33±0.95^a^	18.77±0.39^a^	0.57±0.02^ab^	25.44±1.02^c^
10	75.32±0.26^b^	62.01±1.02^b^	40.80±1.06^a^	60.45±1.06^a^	42.75±0.75^a^	17.70±0.31^a^	0.54±0.01^ab^	42.69±3.03^b^
20	85.18±1.42^a^	72.37±1.45^a^	40.31±1.05^a^	61.91±1.46^a^	43.78±1.03^a^	18.13±0.43^a^	0.60±0.03^a^	57.20±7.62^a^
30	67.02±0.93^c^	51.80±1.07^c^	36.37±0.71^b^	54.65±0.77^b^	38.64±0.55^b^	16.01±0.23^b^	0.56±0.01^ab^	32.55±0.15^bc^
40	32.95±1.27^d^	21.80±0.38^e^	35.00±1.74^b^	50.48±3.11^bc^	35.70±2.20^bc^	14.79±0.91^bc^	0.50±0.03^b^	14.15±1.17^d^
50	23.81±0.92^e^	16.00±0.73^f^	33.55±0.85^b^	47.78±1.54^c^	33.79±1.08^c^	13.99±0.45^c^	0.41±0.02^c^	10.82±0.40^d^

EY, egg yolk; TM, total motility; PM, progressive motility; VSL, straight-line velocity; VCL, curvilinear velocity; VAP, average path velocity; ALH, amplitude of lateral head displacement; WOB, wobble movement coefficient; MAD, average motion degree.

a–f Means within a column with different superscripts differ significantly (p<0.05).
